# Decoding the interplay between COVID-19 and diabetic nephropathy through bioinformatics and systems biology techniques

**DOI:** 10.1016/j.bbrep.2025.102366

**Published:** 2025-11-17

**Authors:** Xianxiang Chen, Qingle Zeng, Min Xia, Yufen Chen

**Affiliations:** a Department of Colorectal Surgery, Sun Yat-sen University Cancer Center, State Key Laboratory of Oncology in South China, Guangzhou, China; b Department of Nutrition, School of Public Health, Sun Yat-sen University, Guangzhou, China; c The First Clinical Medical College, Guangxi Medical University, Nanning, China; d Health Management Center, People's Hospital of Guangxi Zhuang Autonomous Region and Guangxi Academy of Medical Sciences, Nanning, China

**Keywords:** COVID-19, Diabetic nephropathy, Differentially expressed genes, Regulatory network, Pathogenic mechanisms

## Abstract

**Aims:**

Individuals with diabetic nephropathy (DN), a major diabetic complication, have been disproportionately affected by the coronavirus disease 2019 (COVID-19) pandemic. This study aimed to investigate the molecular interplay between COVID-19 and DN using bioinformatics and systems biology approaches to identify shared mechanisms and therapeutic targets for their improved synergistic clinical management.

**Methods:**

Transcriptomic datasets (COVID-19, GSE171110; DN, GSE30528) were analyzed to identify differentially expressed genes (DEGs). Additionally, functional enrichment, protein-protein interaction (PPI) networks, transcription factor (TF)–microRNA (miRNA) regulatory networks, and drug-gene associations were explored. The diagnostic potential of hub genes was validated using receiver operating characteristic curves.

**Results:**

In total, 3975 DEGs (2796 upregulated; 1179 downregulated) were identified in patients with COVID-19 versus controls, and 348 DEGs (93 upregulated; 255 downregulated) were found in patients with DN. Among them, 83 DEGs overlapped, presenting shared molecular pathways, including hematopoietic cell lineage, focal adhesion, and complement/coagulation cascades. PPI analysis revealed five major hub genes (*IL7R*, *CD2*, *GZMA*, *CD3D*, and *FCER1A*) associated with immune regulation and tissue injury, and regulatory network analysis identified 46 TFs and 88 miRNAs interacting with them. Based on transcriptomic signatures, drug repurposing candidates, such as alpha-d-mannose, aspirin, and methotrexate, were identified. Additionally, hub genes showed a high diagnostic potential (area under the curve >0.80 for COVID-19 and DN). Finally, we use external datasets to validate hub genes.

**Conclusions:**

The findings of this study reveal shared molecular pathways and hub genes between COVID-19 and DN, providing insights into immune dysregulation and tissue injury mechanisms. Strategies associated with identified biomarkers and therapeutic candidates, including interleukin-7 receptor-targeting strategies, offer the potential for improving clinical outcomes in patients with comorbid COVID-19 and DN. Lastly, these findings underscore the value of integrative bioinformatics in guiding precision medicine approaches for complex disease interactions.

## Highlights

• Transcriptomics found 83 shared DEGs and converging pathways in COVID-19 vs diabetic nephropathy, driving immune dysregulation and renal injury.
• PPI-identified hub genes IL7R/CD2/GZMA/CD3D/FCER1A diagnose COVID-19 and diabetic nephropathy with AUC>0.80, linking immune activation to tissue damage.
• IL7R modulation and repurposed drugs (α-d-mannose, aspirin, methotrexate) validated via TF/miRNA networks offer new therapeutic targets.
• Complement overactivation drives COVID-19/diabetic-nephropathy crosstalk, worsening renal pathology and thrombosis.
• Multi-omics systems model guides precision combo therapy for COVID-19 plus diabetic nephropathy.

## 1 Introduction

The coronavirus disease 2019 (COVID-19) pandemic has substantially affected patients with diabetes, garnering considerable attention worldwide [1,2]. Diabetic nephropathy (DN) is a common complication of diabetes and severely affects the quality of life of patients, markedly increasing the risk of cardiovascular diseases and mortality [3,4]. Moreover, the high prevalence of DN imposes a substantial economic burden on both the patients and society [5,6]. Presently, many therapeutic approaches (including blood sugar control, antihypertensive medications, and dietary interventions) are employed to manage DN; however, their efficacy remains limited, especially in the late stages where reversal is not possible [7]. Consequently, there is an urgent need for novel therapeutic strategies to improve the prognosis of patients with DN.

Reportedly, patients with diabetes who contracted COVID-19 were more likely to experience severe complications [8,9], suggesting a potential interaction between COVID-19 and diabetes. COVID-19 has been shown to exacerbate DN progression by triggering inflammatory and immune responses [10,11], providing novel insights into the COVID-19–DN relationship. However, the specific mechanisms underlying COVID-19–DN interaction remain unelucidated, indicating a notable research gap. Hence, this study aimed to employ bioinformatics and systems biology approaches to analyze COVID-19- and DN-related gene expression data and biological pathways. This approach provides the advantage of integrating multiple data sources to identify complex biological relationships and potential mechanisms. For instance, COVID-19 infection has been associated with aggravated kidney damage in patients with diabetes by regulating the expression of certain key genes, such as *ACE2*, *IL6*, and *TNF-α* [12].

**Fig. 1:**
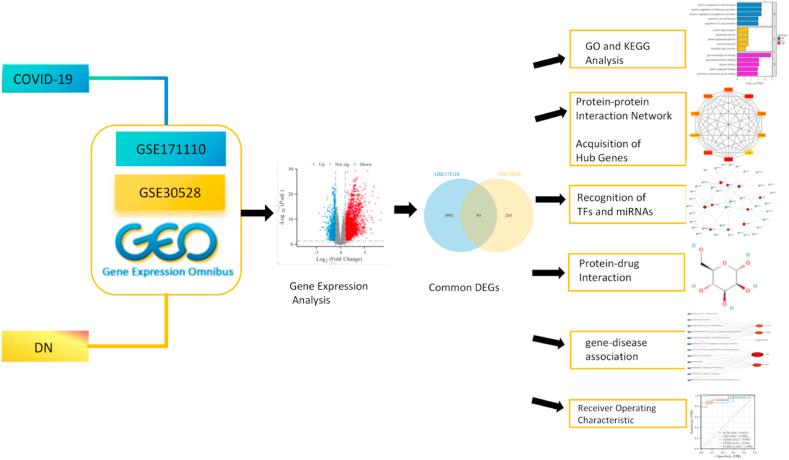
The overall work of this study.

Herein, the mechanisms underlying the interactions of COVID-19 and DN, along with potential therapeutic targets, were explored to provide scientific evidence for developing synergistic treatment approaches for COVID-19 and DN in clinical settings. The integrated findings of bioinformatics and systems biology techniques obtained in this study may offer new perspectives for the combined clinical management of COVID-19 and diabetes and provide a theoretical basis for developing related drugs in the future and advancing research in related fields. Fig. 1 provides a detailed depiction of the study's specifc methodology.

## 2 Materials and methods

### 2.1 Datasets

The transcriptomic datasets GSE171110 and GSE30528 were obtained from the Gene Expression Omnibus (GEO) database (https://www.ncbi.nlm.nih.gov/geo/) for COVID-19 and DN, respectively [13]. The GSE171110 dataset, constructed on the GPL16791 platform, contains data from 44 patients with COVID-19 and 10 healthy subjects. The GSE30528 dataset, created on the GPL571 platform, includes the data from 9 DN tissue samples and 13 control samples. Table 1 presents the basic information for both datasets.

**Table 1:** Overview of the datasets used in this study along with their GEO-features and quantitative measurements.

Disease name	GEO accession	GEO platform	Total DEGs count	Up regulated DEGs count	Down regulated DEGs count
COVID-19	GSE171110	GPL16791	3975	2796	1179
DN	GSE30528	GPL571	348	93	255

### 2.2 Identification of differentially expressed genes (DEGs) common between COVID-19 and DN datasets

To identify DEGs between COVID-19 and control samples in the GSE171110 dataset, the limma package within the R programming language (version 4.2.1) was employed [14]. The Benjamini–Hochberg false discovery rate (FDR) method was used to identify statistically significant genes while controlling for false positives. Ultimately, genes meeting the following threshold criteria: adjusted P-value <0.05 and absolute log2 fold change (|log2FC|) ≥ 1.0 were classified as DEGs. The same analytical pipeline was applied to identify the DEGs between DN and control samples in the GSE30528 dataset. Additionally, volcano plots and heatmaps were generated using the ggplot2 and ComplexHeatmap packages, respectively. To identify intersecting DEGs between the GSE171110 and GSE30528 datasets, the VennDiagram package in R was employed.

### 2.3 Functional enrichment analysis of common DEGs

Gene ontology [15] (GO) and Kyoto Encyclopedia of Genes and Genomes [16] (KEGG) pathway enrichment analyses were performed to investigate DEG-enriched biological activities and pathways. The functional characteristics of DEGs common between COVID-19 and DN datasets, GO and KEGG enrichment analyses were performed using the clusterProfiler package in R [17], with p-value <0.05 set as the significance threshold.

### 2.4 Protein–protein interaction (PPI) network analysis and hub gene prediction

PPI network analysis is crucial for assessing cellular biochemical reaction networks to obtain functional and structural insights. Herein, a systematic evaluation of PPI networks based on shared DEGs was conducted using the Search Tool for the Retrieval of Interacting Genes/Proteins (STRING) database (version 12.0) [18]. PPI networks were constructed and visualized with Cytoscape (v3.10.3) [19], with a combined interaction score threshold set at >0.15. Subsequently, the most interconnected nodes in the PPI network, denoting hub genes, were identified. The key genes within PPI network modules were ranked, analyzed, and identified using the cytoHubba plugin in Cytoscape [20]. The top five hub genes in the PPI network were selected using the Maximal Clique Centrality (MCC) method.

### 2.5 Identification of transcription factors (TFs) and microRNAs (miRNAs)

The transcriptional landscape and critical regulators governing shared DEGs were explored by comprehensively investigating the integrated DEG–miRNA and DEG–TF interactions. Statistical analysis, visualization, and meta-analysis of the network-based gene expression data were performed using the NetworkAnalyst platform (https://www.networkanalyst.ca/) [21]. The NetworkAnalyst, a widely used online tool, enables the identification of structurally robust TFs from the JASPAR database, consisting of comprehensive multi-species TF-binding profiles [22]. Experimentally validated miRNA–target interaction data were sourced from the microRNA–target interactions database and the miRNA targets database [23,24]. The aforementioned databases were accessed to construct separate networks for DEG–miRNA and DEG–TF interactions. Subsequently, overlapping regions between the two networks were identified to highlight their structural significance, with particular emphasis on DEG-associated miRNAs.

### 2.6 Analysis of gene-disease interaction networks

Exploring genetic information underlying human diseases represents a cornerstone of precision medicine and drug discovery [25]. Disease-centric Gene and Variant Network (DisGeNET), a knowledge management platform, aggregates and standardizes data from multiple genetic and disease variant databases [26]. Herein, the NetworkAnalyst platform was used to identify gene-disease relationships, revealing disease associations linked to both COVID-19 and DN.

### 2.7 Evaluation of potential drugs

The Drug Signature Database (DSigDB), containing 22,527 gene sets [27], was employed to identify small compounds capable of downregulating the identified hub genes. The DSigDB library was accessed through the Enrichr platform (https://amp.pharm.mssm.edu/Enrichr/) to identify drug entities based on the common DEGs [28]. Subsequently, potential pharmacological molecules influencing key gene expression were systematically identified. The findings of this systematic evaluation may provide insights for developing targeted therapeutic interventions.

### 2.8 Diagnostic value of hub genes

The diagnostic performance of hub genes for COVID-19 and DN was evaluated by constructing receiver operating characteristic (ROC) curves and calculating the area under the curve (AUC) using the pROC R package [29].

### 2.9 Validation of hub genes in external datasets

In order to minimize false-positive findings, we further validated the identified hub genes using supplementary datasets for COVID-19 and DN from the GEO database. For the COVID-19 analysis, we used datasets GSE152418, obtaining 17 COVID-19 cases and 17 healthy controls. In parallel, for the DN study, we used datasets GSE104948, resulting in a pooled set of 7 cirrhotic tissue samples and 18 control samples. To ensure rigorous statistical analysis, the Welch *t*-test (implemented via the stats and car packages) was used for hypothesis testing, while data visualization was carried out with the ggplot2 package.

### 2.10 Statistical analysis

All statistical analyses were performed using R software (version 4.2.1). For the identification of DEGs in the COVID-19 (GSE171110) and DN (GSE30528) datasets, the limma R package was employed. The Benjamini-Hochberg false discovery rate (FDR) method was applied to adjust for multiple comparisons, and genes with an adjusted p-value <0.05 and an absolute log2 fold change (|log2FC|) ≥ 1.0 were considered statistically significant DEGs. No missing data were present in the analyzed datasets as provided by GEO, and thus no imputation was necessary. Functional enrichment analysis of GO terms and KEGG pathways was conducted using the clusterProfiler package, with a significance threshold of p-value <0.05. For the validation of hub genes in external datasets (GSE104948 for DN and GSE152418 for COVID-19), the Welch's *t*-test (a variant of the Student's t-test that does not assume equal variances) was used to compare gene expression levels between disease and control groups. This test was implemented using the stats and car packages in R. Statistical significance in the validation figures is denoted as follows: ∗p < 0.05, ∗∗p < 0.01, ∗∗∗p < 0.001; ns, not significant (p > 0.05).

## 3 Results

### 3.1 Identification of DEGs and common DEGs between COVID-19 and DN

**Fig. 2:**
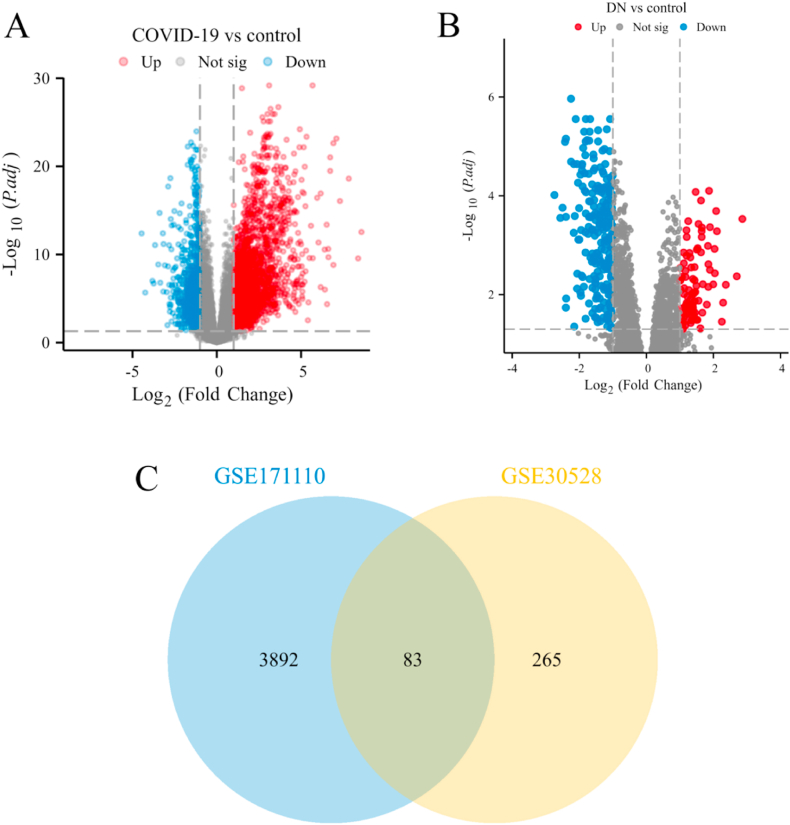
Volcano plots exhibit DEGs of (A)COVID-19, (B)DN. (C) The Venn diagram depicts the common DEGs among COVID-19 and DN.

To investigate the interplay between COVID-19 and DN, human RNA sequencing (RNA-seq) datasets were collected and analyzed. In total, 3975 DEGs were identified between patients with COVID-19 and healthy controls in the GSE171110 dataset, including 2796 upregulated and 1179 downregulated DEGs. Similarly, 348 DEGs were detected between DN tissues and control samples in the GSE30528 dataset, with 93 upregulated and 255 downregulated DEGs (Table 1). Volcano plots show the transcriptional profiles of COVID-19 and DN, with red and blue dots denoting significantly upregulated and downregulated genes, respectively (Fig. 2A and B). In total, 83 shared DEGs were identified between GSE171110 (COVID-19) and GSE30528 (DN) datasets (Fig. 2C), suggesting mechanistic overlaps between the two diseases.

### 3.2 GO and KEGG pathway enrichment analysis

**Fig. 3:**
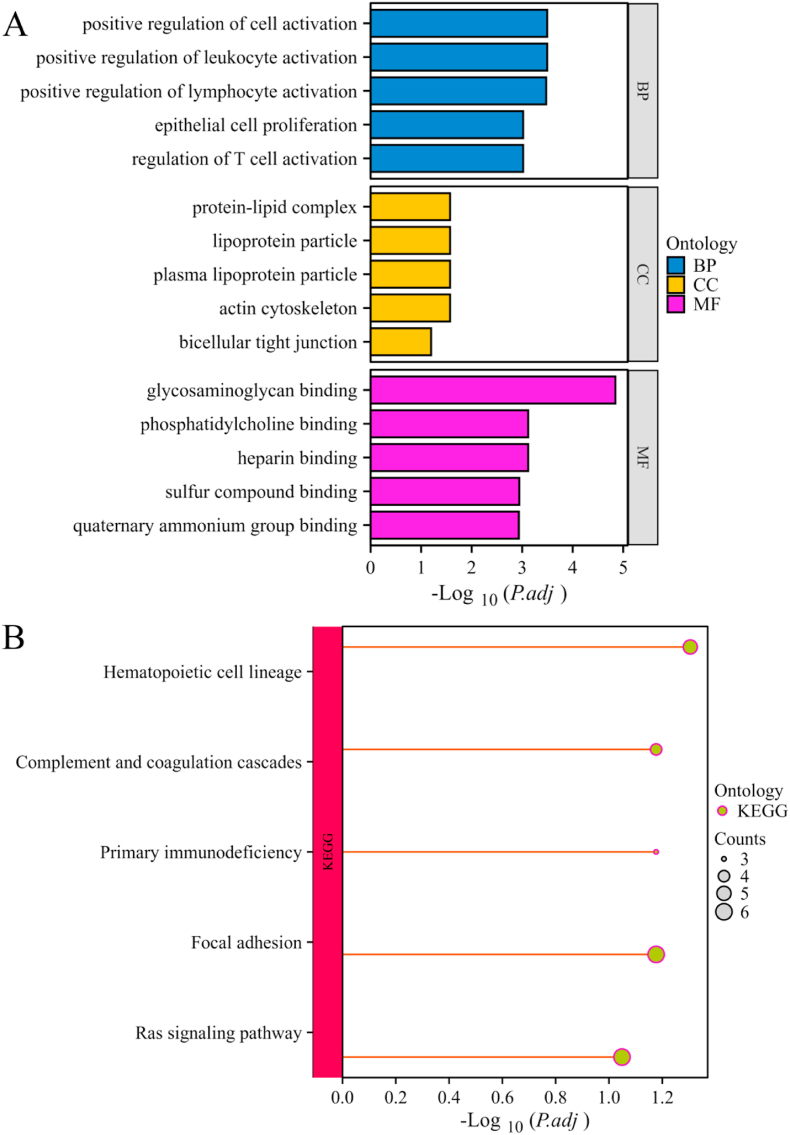
(A) The bar graphs of the ontological analysis of the common DEGs among COVID-19 and DN. (B) Bubble graphs indicate the results for KEGG analysis based on the common DEGs among COVID-19 and DN.

GO analysis revealed DEG-associated enriched biological processes, cellular components, and molecular functions. The top five enriched terms in each category are presented in Fig. 3A. KEGG pathway analysis revealed that the following pathways were most significantly enriched: hematopoietic cell lineage, focal adhesion, primary immunodeficiency, complement and coagulation cascades, and Ras signaling pathway (Fig. 3B).

### 3.3 PPI network construction and hub gene identification

**Fig. 4:**
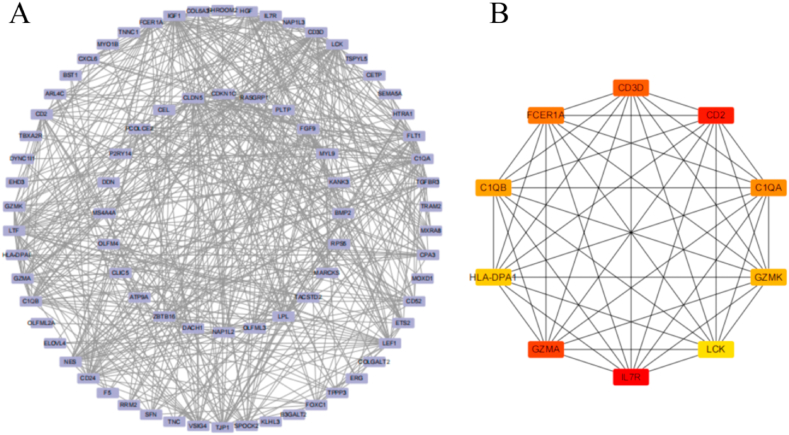
Development of PPI network and extraction of hub genes (A) The visualization of the PPI network of shared DEGs was enabled by Cytoscape.(B) The top ten hub gene modules identified by MCC function of Cytohubba.

The PPI network was constructed using the STRING database based on the 83 shared DEGs, which yielded 78 nodes and 520 edges (Fig. 4A). The CytoHubba plugin in Cytoscape identified the following top 10 hub genes via the MCC algorithm: *IL7R*, *CD2*, *GZMA*, *CD3D*, *FCER1A*, *C1QA*, *C1QB*, *GZMK*, *HLA-DPA1*, and *LCK* (Fig. 4B).

### 3.4 Identification of hub gene-associated TFs and miRNAs

**Fig. 5:**
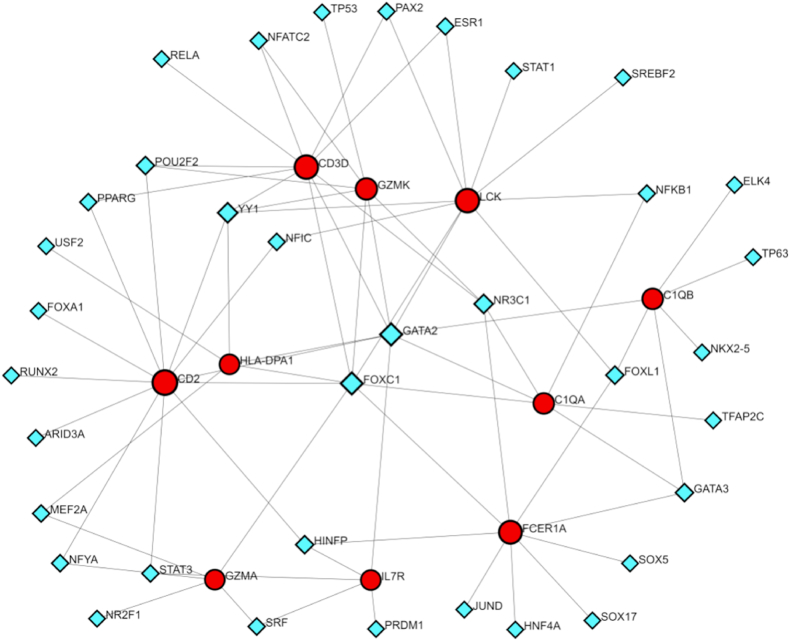
Construction of Regulatory Networks. Interaction network between TFs and DEGs. The network uses blue nodes to symbolize TFs, red nodes for DEGs, and directional arrows to indicate regulatory relationships.

**Fig. 6:**
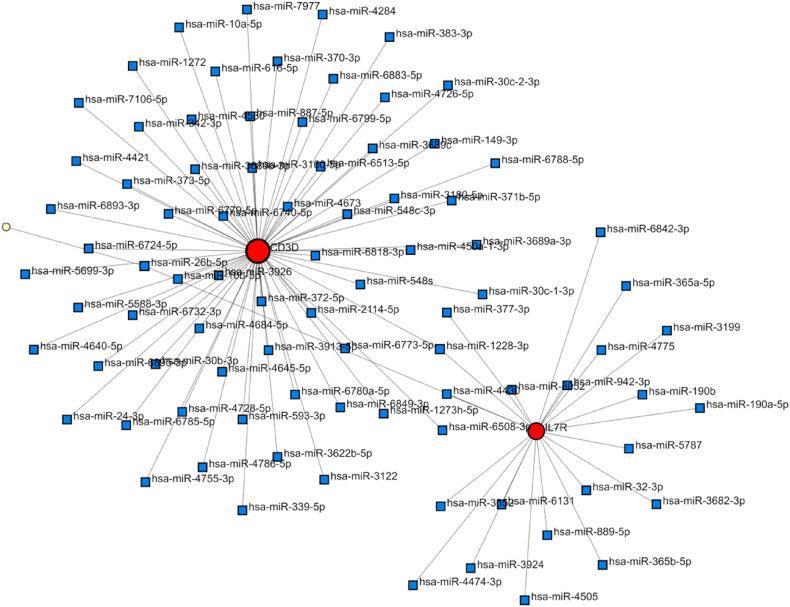
Construction of Regulatory Networks. (B) The network of gene-miRNA interactions. The blue color nodes represent the miRNAs and the other color nodes represent hub genes.

The NetworkAnalyst identified 46 TFs and 88 miRNAs regulating the hub genes (Fig. 5, Fig. 6), highlighting potential upstream mechanisms driving the expression of shared DEGs.

### 3.5 Identification of disease association

**Fig. 7:**
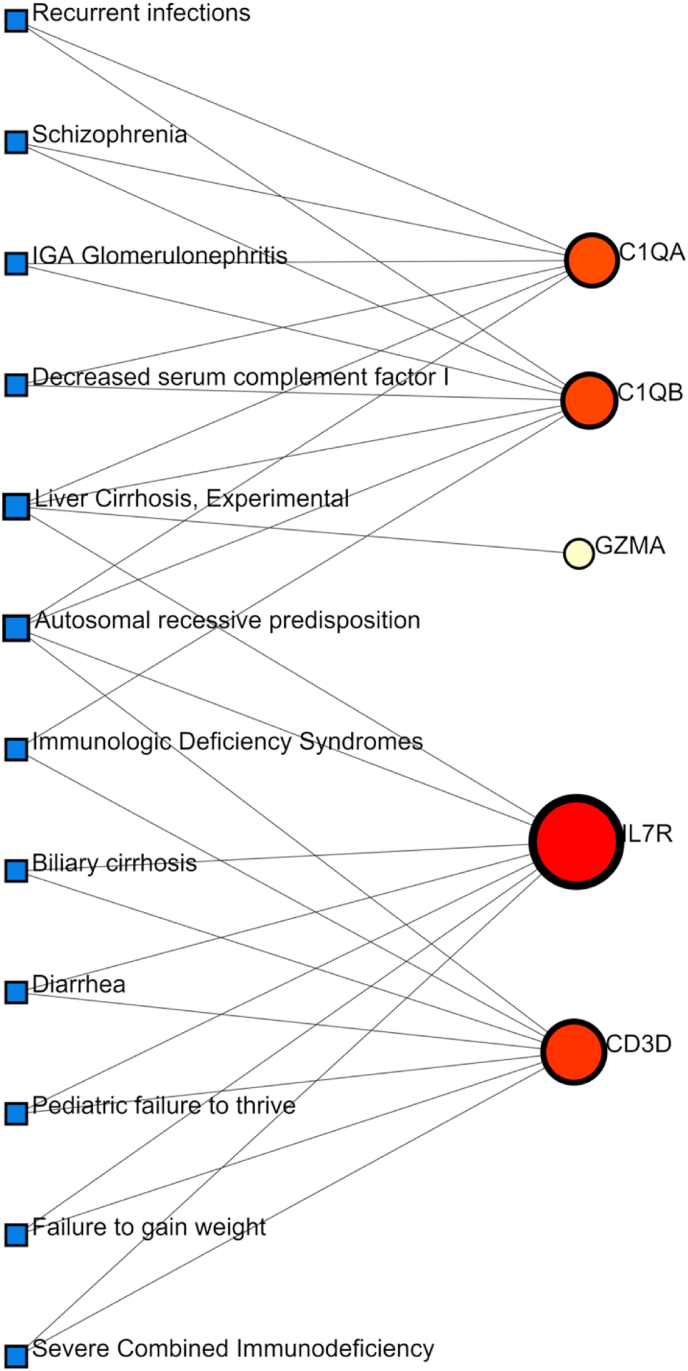
The gene-disease association network represents diseases associated with common DEGs. The disorders depicted by the square node and also its subsequent gene symbols are defined by the circular node.

Disease*–*gene association analysis using DisGeNET linked the hub genes to the following conditions: autosomal recessive predisposition, liver cirrhosis, immunologic deficiency syndromes, diarrhea, and biliary cirrhosis (Fig. 7), underscoring their clinical relevance.

### 3.6 Identification of candidate drugs

Enrichr/DSigDB analysis revealed the 10 candidate drug molecules targeting the hub genes, including alpha-d-Mannose, dichloroacetate, ZIRAM, aspirin, ephedrone, methotrexate, (−)-isoprenaline, saracatinib, 3,3′,4,4′,5-pentachlorobiphenyl, and Go 6976 (Fig. 9).

**Fig. 8:**
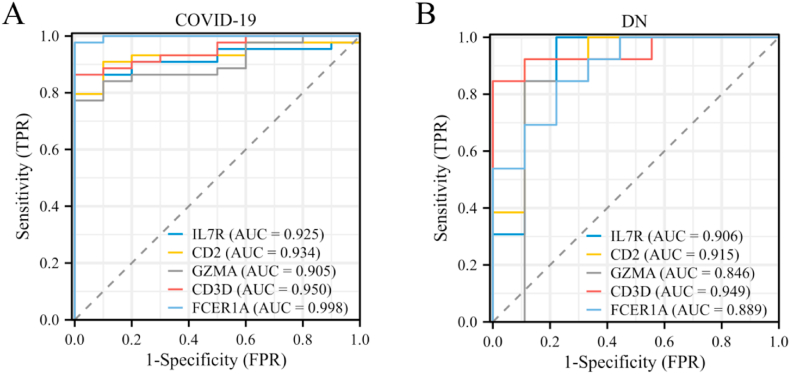
Assessment of hub genes in diagnostic value. (A) The diagnostic efficacy verification in COVID-19. (B) The diagnostic efficacy verification in DN.

**Fig. 9:**
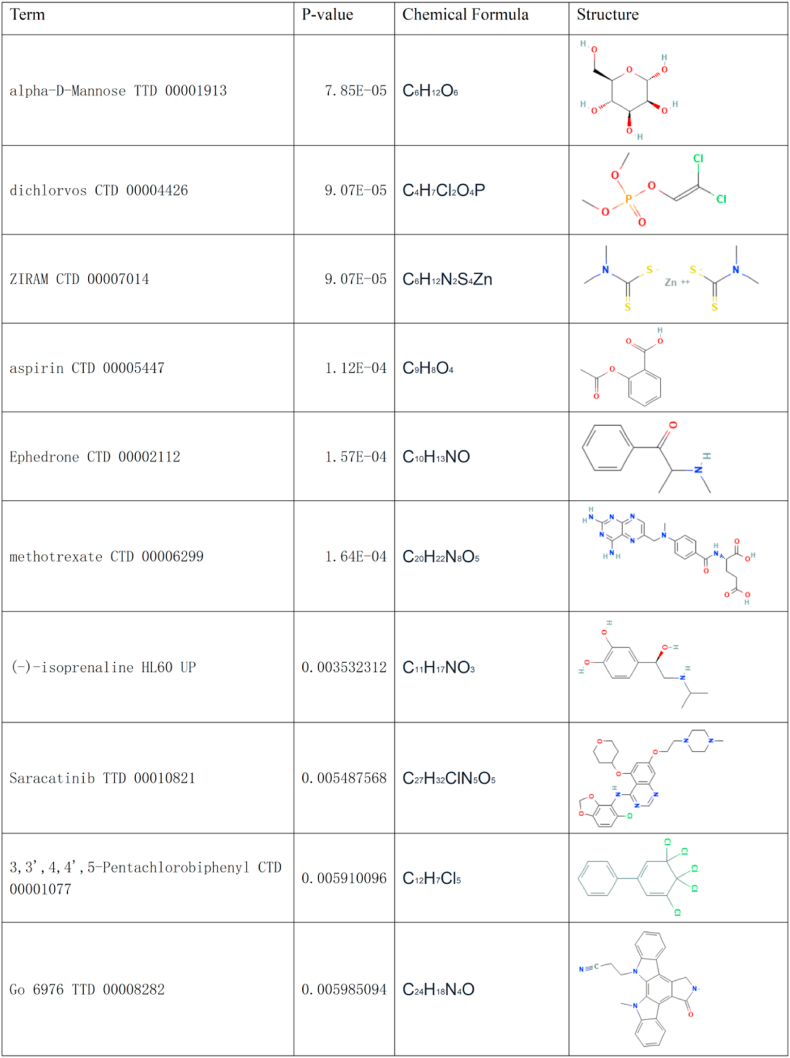
The recommended drugs for COVID-19 and DN.

### 3.7 Assessment of diagnostic values of hub genes

ROC analysis revealed strong diagnostic performance of five hub genes based on their AUC values. For COVID-19, the AUCs of *IL7R*, *CD2*, *GZMA*, *CD3D*, and *FCER1A* were 0.925, 0.934, 0.905, 0.950, and 0.998, respectively (Fig. 8A). For DN, the AUCs of *IL7R*, *CD2*, *GZMA*, *CD3D*, and *FCER1A* were 0.906, 0.915, 0.846, 0.949, and 0.889, respectively (Fig. 8B). These results highlight their potential as biomarkers for both diseases.

### 3.8 Validation of hub genes in external datasets

**Fig. 10:**
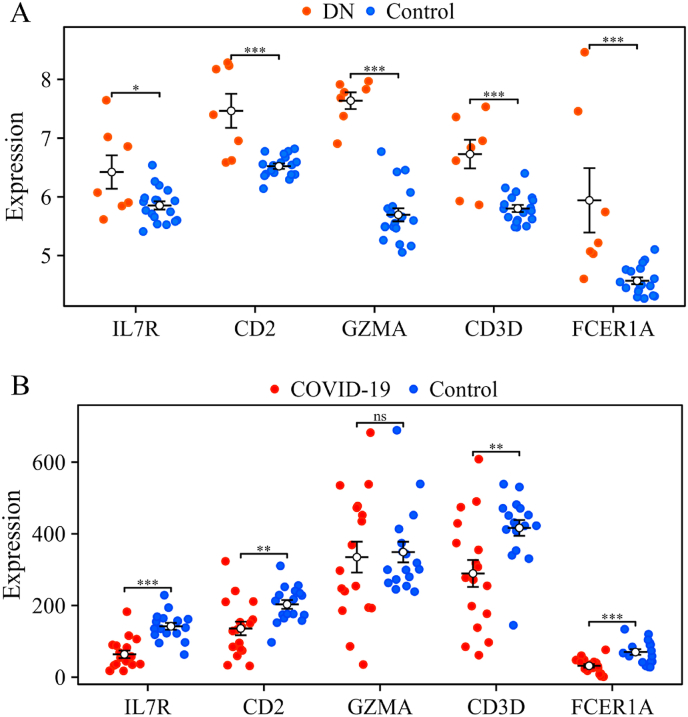
Validation of the hub genes in the external datasets. (A) The expression of IL7R, CD2, GZMA, CD3D and FCER1A in the diabetic nephropathy dataset GSE104948. (B) The expression of IL7R, CD2, GZMA, CD3D and FCER1A in the COVID-19 dataset GSE152418. ∗p < 0.05, ∗∗p < 0.01, ∗∗∗p < 0.001; ns: p > 0.05, based on Welch’ s *t*-test.

To assess the robustness of the five hub genes, we first examined their expression profiles using the dataset from GSE104948. As illustrated in Fig. 10A, the expression levels of IL7R, CD2, GZMA, CD3D and FCER1A significantly differed between diabetic nephropathy and controls. In a parallel analysis, the datasets from GSE152418 revealed pronounced differences in the expression of IL7R, CD2, CD3D and FCER1A when comparing COVID-19 samples to control samples (Fig. 10B).

## 4 Discussion

The emergence of COVID-19, caused by the novel severe acute respiratory syndrome coronavirus 2 (SARS-CoV-2) and manifesting as severe respiratory illness and various systemic complications, triggered a global health crisis [30]. COVID-19 could exacerbate the condition of patients with diabetes [1]. DN is a major complication of diabetes, leading to progressive kidney damage and increased morbidity and mortality [31]. Consequently, different studies showed associations between complex pathophysiological mechanisms of COVID-19 and DN, including inflammation and immune dysregulation, suggesting common molecular pathways [32]. Owing to patients with diabetes and COVID-19 presenting an increased risk of severe outcomes, understanding COVID-19–DN interactions is crucial [33,34]. Hence, this study aimed to shed light on DEG profiles and potential shared mechanisms between these two diseases, thereby contributing to the understanding of their interrelationship and providing a research basis for developing treatment strategies.

Herein, identifying key genes was critical for understanding COVID-19–DN interactions. Among the identified central genes, *IL7R* was of the highest significance, possibly owing to its key role in regulating immune responses. Reportedly, the interleukin-7 receptor (IL7R) is essential for the development and homeostasis of T cells, and its dysregulation has been associated with various autoimmune diseases and infections [[35], [36], [37]]. IL7R upregulation in both COVID-19 and DN suggests a possible connection between immune dysregulation and kidney injury, showing its importance as a marker regarding disease severity and progression. Hence, targeting IL7R may provide therapeutic avenues for modulating immune responses in both these diseases. *CD2*, encoding cluster of differentiation 2 (CD2) and another key gene identified in this study, is known for its role in T cell activation and adhesion [38]. As a co-stimulatory molecule, CD2 enhances T cell responses [39], which is critical for alleviating viral infections such as COVID-19. Herein, upregulated CD2 indicated an enhanced immune response, which may impact the pathophysiology of both COVID-19 and DN. The dual role of *CD2* in promoting immune activation and potentially participating in managing kidney inflammation highlights its importance as a therapeutic target. These findings suggest that inhibiting CD2 signaling could mitigate excessive immune responses, thereby protecting kidney function in patients with both COVID-19 and DN. Lastly, *GZMA*, encoding granzyme A(GZMA), is another significant key gene identified in this study. GMZA is a serine protease that plays a critical role in the cytotoxic activity of T and natural killer cells [[40], [41], [42]]. GZMA upregulation in our study aligns with the findings of previous studies associating GZMA with tissue damage and inflammation in various diseases. GZMA involvement in both COVID-19 and DN indicates a potential shared pathway for immune-mediated tissue damage, which may exacerbate kidney damage in patients with COVID-19. Understanding the role of GZMA in these diseases may lead to the development of novel therapeutic strategies aimed at reducing tissue damage while preserving immune function. Overall, these central genes provide insights into the shared molecular pathways between COVID-19 and DN and serve as potential therapeutic intervention targets. Our findings are consistent with previous research strategies that utilized similar bioinformatics methods to identify key genes and drugs for COVID-19 [43].

Pathway enrichment analysis revealed many key biological pathways that may serve as the potential mechanisms connecting COVID-19 and DN, with the “hematopoietic cell lineage” pathway being the most significant contributor. This hematopoietic cell lineage pathway is crucial for the development and differentiation of blood cells, which, in turn, play a key role in immune responses [[44], [45], [46]]. Hence, its dysregulation may lead to the abnormal immune responses observed in patients with COVID-19, which potentially exacerbate renal complications in patients with pre-existing DN. Alterations in hematopoietic cell function have been reported to affect the severity of viral infections [47,48], suggesting it as a potential therapeutic avenue for managing comorbidities. Another prominent pathway identified in this study is “focal adhesion,” which plays a critical role in cell signaling and tissue structure maintenance [49]. Dynamic changes in focal adhesion are essential for cellular responses to external stimuli, including inflammatory signals [50,51]. Reportedly, SARS-CoV-2 can trigger notable inflammatory responses, disrupting normal cell adhesion processes and leading to tissue damage [52]. Additionally, altered focal adhesion signaling in DN has been shown to contribute to glomerular injury and fibrosis [53]. The understanding of these pathway interactions regarding COVID-19 and DN may facilitate formulating strategies to mitigate kidney damage during viral infections. Additionally, “complement and coagulation cascades” was identified to be a significantly enriched pathway. This pathway is central to immune responses and associated with the pathophysiologies of both COVID-19 and DN [54,55]. In COVID-19, excessive activation of the complement system has been associated with severe disease outcomes, including acute respiratory distress syndrome [54,56]. Moreover, in DN, complement activation leads to glomerular inflammation and injury [55]. The intersection of these pathway outcomes indicates complement and coagulation cascades as a potential therapeutic target, suggesting that modulating complement activity may alleviate both virus-induced and DN complications. Overall, these pathway analyses emphasize the complex interactions between COVID-19 and DN, underscoring the need for integrated therapeutic approaches to address both diseases simultaneously.

The identification of potential drug molecules based on DEGs associated with COVID-19 and DN provides a promising avenue for therapeutic intervention. Among the 10 candidate drugs identified in this study, α-d-mannose is of notable significance owing to its role in modulating immune responses and its potential to enhance the efficacy of antiviral therapies [57]. Reportedly, α-d-mannose can inhibit viral adhesion [58], thereby reducing infection severity, which is particularly relevant regarding COVID-19. This antiviral property of α-d-mannose presents it as a beneficial compound for managing patients with concurrent COVID-19 and DN, considering its ability to mitigate viral infection-associated exacerbation of kidney complications. Dichloroacetate, an organophosphorus compound, has been recognized for its anti-inflammatory properties [59]; consequently, its application in COVID-19 management is of significance [60], considering the excessive inflammatory responses observed in severe COVID-19 cases. Reportedly, dichloroacetate can reduce the production of inflammatory cytokines, which is crucial for preventing the progression of both COVID-19 and DN [60,61]. This underscores its potential as a therapeutic agent, warranting further investigation, especially in patients exhibiting overlapping pathophysiological features of both diseases. Aspirin, a well-established anti-inflammatory and antiplatelet drug, has garnered notable attention for its potential in managing COVID-19 [62]. Moreover, recent studies have highlighted its ability to reduce thrombotic events [63], which are common in severe COVID-19 cases. Additionally, aspirin has been shown to alleviate the risk of cardiovascular complications in patients with DN [64], making it a valuable candidate for further exploration regarding the management of both COVID-19 and DN. Ephedrine, a sympathomimetic drug, can enhance respiratory function and alleviate symptoms of viral infections [65], indicating its potential role in modulating immune responses may be particularly beneficial for patients with COVID-19, especially those with DN-associated impaired kidney function. The therapeutic significance of ephedrine warrants further clinical evaluation regarding its therapeutic efficacy for both diseases. Methotrexate, a well-known immunosuppressant, has been used in the management of various inflammatory diseases [66]. It can modulate immune responses, suggesting its potential application in COVID-19 treatment, which is under investigation. In patients with DN, methotrexate may help control inflammation, thereby reducing the risk of COVID-19-associated acute kidney injury. Overall, these identified candidate drugs show the potential for repurposing to address the dual challenges of COVID-19 and DN. Future research should focus on elucidating the mechanisms of action of these drugs regarding both diseases, paving the way for innovative therapeutic strategies that can substantially improve patient care.

The methodology employed in this study offers many advantages that enhance the robustness and reliability of the findings. First, established RNA-seq datasets from the GEO database were used, namely GSE171110 and GSE30528, which ensured that the analysis was based on high-quality publicly available data, facilitating further reproducibility and validation. The DEG analysis using the limma package, combined with the Benjamini–Hochberg method to control the FDR, presented a rigorous statistical approach that minimized the likelihood of false positives. Furthermore, GO and KEGG functional enrichment analysis provided a comprehensive understanding of the biological significance of the identified DEGs. The constructed PPI networks using STRING and hub genes identified using the MCC method in Cytoscape elucidated the complex interactions between proteins and highlighted potential key regulatory factors regarding COVID-19 and DN. Finally, the inclusion of TF and miRNA analyses, along with gene-disease interaction networks, enriched the interpretative framework of the present study, providing a robust basis for future therapeutic explorations. The comprehensive analytical process we adopted, including the exploration of the non-coding RNA regulatory network, has been proven to be highly effective in revealing prognostic biomarkers and mechanisms of complex diseases such as colorectal cancer [67] and gastric adenocarcinoma [68]. Moreover, the in-depth understanding of transcriptional regulation can be achieved through advanced motif detection tools [69], which points the way for future research on the shared transcriptional regulatory circuits between COVID-19 and DN. Overall, these methodological advantages contribute to a deeper understanding of the interactions between COVID-19 and DN for further advances in the field of precision medicine.

## 5 Conclusion

The findings of this study elucidate the complex interactions between COVID-19 and DN through a comprehensive analysis of RNA-seq datasets. DEGs identified in this study showed significant overlaps, indicating shared molecular pathways and potential common mechanisms between the two diseases. Functional enrichment analysis further highlighted key BPs and pathways, such as hematopoietic cell lineage and focal adhesion, which might underlie the pathogenesis of both diseases. Notably, PPI network analysis identified critical hub genes, including *IL7R* and *CD2*, which exhibit strong diagnostic potential, as evidenced by their high AUC values. Additionally, the integration of TF and miRNA analysis provided insights into the regulatory networks governing the identified DEGs. The identification of candidate drugs through the DSigDB database emphasized the therapeutic possibilities targeting the shared pathways between COVID-19 and DN. Overall, this study enhances the understanding of the molecular links between COVID-19 and DN and provides a theoretical basis for future research to develop targeted treatments.

## Author contributions

Yufen Chen and Min Xia conceived and designed the study. Yufen Chen, Xianxiang Chen, Qingle Zeng and Min Xia involved in data acquisition, analysis, and interpretation. Xianxiang Chen and Qingle Zeng wrote the final manuscript. All authors have read and approved the final version of the manuscript.

## Ethics statement

Not Applicable.

## Declaration of competing interest

The authors declare no competing interests.

## Data Availability

The datasets presented in this study can be found in online repositories. The names of the repository/repositories and accession number(s) can be found below: https://www.ncbi.nlm.nih.gov/geo/
